# Teaching Epistemology – Workshop as a Method for making Epistemology Relevant to Students

**DOI:** 10.15694/mep.2021.000140.1

**Published:** 2021-05-22

**Authors:** Ingrid Jepsen, Lene Toxvig, Annegrethe Nielsen

**Affiliations:** 1 University College Northern Denmark; 2 University College Copenhagen

**Keywords:** health care education, research literacy, epistemology, midwifery, workshop

## Abstract

This article was migrated. The article was marked as recommended.

Introducing epistemology to healthcare students represents a challenge. Yet to be able to practice based on evidence, it is important that healthcare professionals gain an adequate level of research literacy as part of their health care education.

This article presents a new way to teach epistemology using a workshop design.

Three scientific perspectives on epistemology: humanistic, social science and natural science, are used as lenses to analyse situations from everyday healthcare practice represented by a video. Video is used to facilitate transfer of knowledge and to make it meaningful for students to engage in learning epistemology. Afterwards, three teachers facilitate the students’ group work to ensure they get to understand the difference between the scientific perspectives.

The workshop has received very good evaluations as students state that for the first time they understand how epistemology directly links to practice.

## Introduction

Being a contemporary healthcare professional requires competence in seeking, finding, understanding, validating and implementing evidence from scientific literature in order to be able to deliver evidence-based healthcare service ( Walsh, 2012). But, it has been argued that many healthcare graduates do not have the required skills allowing them to base their practice on evidence ( Heebøll-Nielsen, 2011). Others claim that healthcare professionals in general lack research literacy ( Gaissmaier and Gigerenzer, 2008) and that this impacts healthcare services provided. Epistemology can be very difficult to understand for healthcare students who often find that concepts of epistemology are difficult to relate to clinical practice ( Sonne-Ragans, 2012). For this reason, many textbooks on theory of science, philosophy of science or epistemology try to explain why this knowledge is important and thereby motivate students to engage with the material ( Holm, 2018); ( Darlaston-Jones, 2007); ( Sonne-Ragans, 2012); ( Walsh, 2012). Holm states that future knowledge workers have to work **with** knowledge instead of just **have** knowledge, and that development of this ability is part of higher education ( Holm, 2018). According to Darlaston-Jones, it is necessary to understand the epistemological foundation of research - albeit this is most often not taught to students ( Darlaston-Jones, 2007). Moreover, the International Confederation of Midwives (ICM) and Professor in Midwifery Denis Walsh underline that healthcare professionals serving as midwives must be able to critically assess research results to promote evidence-based practice ( International Confederation of Midwives (ICM), 2018; Walsh, 2012).

According to the Ministerial Order on the Bachelor’s Degree Program of Midwifery in Denmark, the newly educated midwife should master “relevant study and working methods both to search for, assess and interpret empirical evidence, theory and research methods” and be able to “initiate and participate in innovation, development and research work” ( Ministry of Education, 2009). However, in the midwifery program in Northern Denmark, we experienced that novice midwifery students found it difficult to understand and comprehend the meaning of epistemology. They discussed whether epistemology was relevant in an education aiming towards becoming a practicing midwife.

One part of the problem seemed to be that students experienced a conceptual gap between theory and practice. This gap is recognized and discussed by many theoreticians. According to Roth, Mavin and Dekker, different forms of consciousness exist in different activity systems, which leads to a theory/practice gap between the world in which we think and the practical world in which we live ( Roth, Mavin, and Dekker, 2014). The authors show how simulation training of pilots narrows the gap between education, theory and practice. This ability to transfer knowledge from theory to practice is important to pilots; however, it is also important to midwifery students and many other students at a bachelor’s degree level.

To address the problem at the Midwifery Department, we have developed a new way to teach epistemology so that knowledge about epistemology can be transferred to midwifery practice.

## Purpose

The purpose of this article is topresentinsights and experience on how to facilitate teaching and learning to make students gain an understanding of epistemological and methodological approaches and make them understand and appreciate the significance of these approaches for midwifery and for themselves as future midwives. This instructional paper is primarily for educators who teach epistemology to students in healthcare professions.

## Workshop as method - timing and participants

We chose to use the workshop as a teaching design for a variety of reasons. Most importantly, this design allows students to be active and focused, which is considered beneficial for their learning outcomes ( Murdoch-Eaton and Whittle, 2012). Three teachers participate and cooperate: a teacher representing natural science, a teacher representing humanities and a teacher representing social sciences, allowing students to ask questions about differences and similarities within epistemology.

The didactic approach is dialogue-based classroom teaching, including presentations, discussions within the classroom and group work.

### Timing of the workshop

The presented workshop is implemented within the first year of the midwifery education in Aalborg Denmark. However, the workshop has also been conducted in other settings; at an international conference in Oslo, Norway; an Australian Midwifery Conference in Brisbane; a workshop for PhD students in Sydney, Australia; and at a workshop for midwifery students in Stavanger, Norway. The discussions at these workshops reached very different levels of insights, largely mirroring the participants’ academic backgrounds.

### Participants

The midwifery students in Aalborg participate in the workshop during their first semester. The workshop is a starting point for teaching research methods and design during the midwifery program.

During the workshop, the students are divided into nine groups. Therefore, the minimal recommended class size for this kind of workshop is 18 students as this allows two students in each group. We have used this method for class sizes up to 36 students.

## Preparations for the workshop

### Goal for epistemology

To effectively work with the transfer of knowledge to clinical practice, we make sure that the goal for epistemology is described for both the theoretical and the clinical part of the education and that these goals are known - yet maybe not appreciated - by the students.

### Information for teachers/workshop instructors

To prepare students for the workshop, we teach philosophy of science within humanistic, social and natural sciences each in blocks of four 45-minute lessons. Two common assignments have been developed by the teachers for the students to work with in these three teaching sessions. Thus, in humanistic, social and natural sciences students work with: The ICM description of a midwife ( International Confederation of Midwives (ICM), 2018) and the “Recommendations Concerning Antenatal Care” ( Sundhedsstyrelsen, 2013).

More about the content of the lessons in humanistic, social and natural sciences is described below written in imperative for clarity and brevity.

### Introduction of the perspective of natural science

Introduce students to the characteristics of natural sciences in general. Ask students to exemplify how natural sciences influence our daily lives and how they can see the influence of natural sciences in the midwifery program as well as the midwife’s work. Then encourage students to discuss the development of measurability and the question of demarcation as well as the concept of being a neutral observer. Introduce rationalism, positivism and falsifications shortly. Introduce students to what characterizes research methods in natural sciences in general and in the health care sector in particular.

### Introduction of the perspective of humanistic science

Introduce the tradition of humanistic sciences and how humanistic science represents the understanding of other persons, their activities, and their products. Introduce humanistic science as a discipline addressing subjective dimensions of experience, feelings and actions among human beings. Explain to students how the humanistic approach is of vital importance to midwifery to understand the individual’s perspective. Present phenomenology to students as a means of eliciting phenomena as they present themselves to the human subject in an experienced assimilation of the world. Present the hermeneutic tradition in two contexts, namely as an interpreting approach informing methodological thinking or as an approach where hermeneutics represent a certain approach within philosophy of science.

### Introduction of the perspective of social science

Provide students with an understanding of the midwife’s professional role and obligations, how socioeconomics and culture influence the organization of pregnancy, birth and family formations and how midwives in a modern society can support babies and families at risk for maldevelopment.

Introduce the characteristics of a social science perspective. Explain how law and guidelines regulate the midwife’s role, and that it is important that students can recognize all levels of societal influence on their practice. Within the frame of social science, the perspectives of social constructivism and critical theory can be introduced as analytical perspectives.

### Building up research literacy

To build up research literacy the students must read an English research article during their first semester. Therefore, find interesting (English-language) research articles on normal birth and midwifery to motivate students to engage themselves in this task. Students read the article and write a short resume in which they state which midwifery problem the article discussed, what the result of the research was (as stated in the article) and how they could see the results integrated in midwifery practice. In our context, this description should be made in Danish, thus forcing the students to show their understanding of what they have read in English.

This exercise invites all students to read a research article in English during their first semester, thereby opening their eyes to sources of evidence available to them. Especially students with low English reading skills can benefit from being pushed to work independently with this assignment as they may otherwise skip the opportunities to practice their English reading skills.

## Description of the 7 steps in the workshop

**Figure 1:**
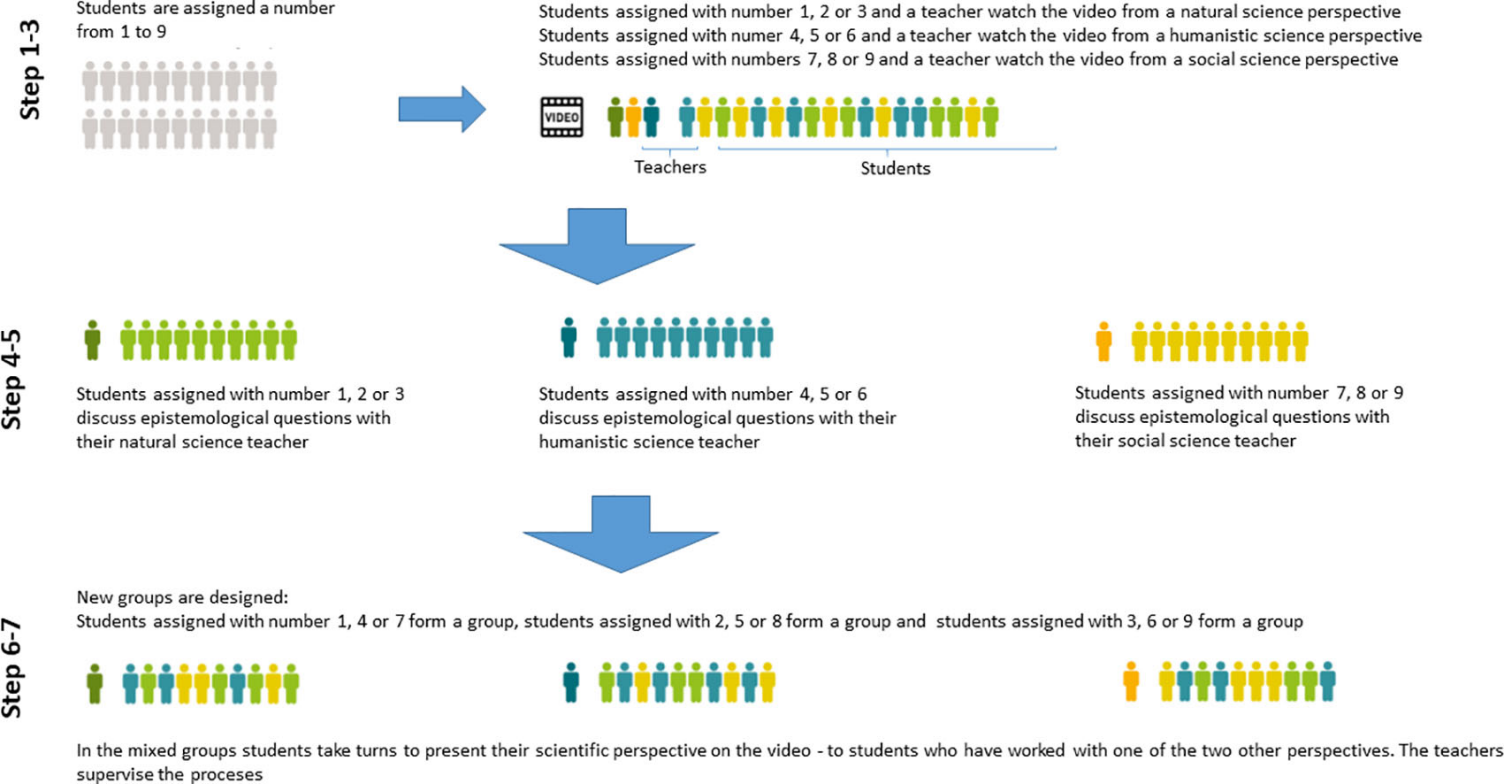
Model of the workshop

The workshop model is shown in Figure 1. In the model, the steps in the workshop are divided into three phases. In Step 1-3, the first phase, the workshop is introduced, the students are assigned numbers and they watch the video. A further explanation of each step is given below. In Step 4-5, the second phase, the students discuss epistemological questions (shown in Table 1) with each other and the teacher and they prepare their presentation. In Step 6-7, the third phase, students from each of the three scientific perspectives present their answers to the epistemological questions and then the workshop is evaluated.

**Table 1:** Questions and guidance for the group work

1) Examples	Give examples from the video of issues that are discussed, treated or mentioned from your assigned perspective of natural, humanistic or social science. Some groups can give concrete examples of actions from their assigned scientific perspective. Other groups may also be required to reflect upon and describe which elements or areas in the video reflect or illustrate their scientific position.
2) Problems	From your scientific position, which questions to the video can be raised? Which problems does the video illustrate? (What could you write about if given an assignment?) Choose one problem you find specifically interesting and typical for your scientific perspective.
3) Scientific perspective	Describe how the problem is typical for your scientific perspective. - And how could working with this problem impact the way midwives work with women giving birth?
4) Method	If you were to explore the problem you have chosen, how would you design a study based on what you know now?

The following description of the 7 steps in the workshop is in imperative to make it short and clear.

### Step 1 - Introduce the workshop design and the participating teachers

Start the workshop by giving a short description of natural, humanistic and social science and the focus of each scientific approach. This facilitates application of the students’ prior learning. Involve all three teachers who taught sciences during the introduction to enhance student’s ability to connect the contents of these lessons to the current workshop. Carefully explain the design of the workshop for the students.

### Step 2 - Assign scientific perspectives to students

Assign students with a number from one to nine. Students numbered 1-3 are instructed to watch the video from the perspective of natural science, that means:" to put on their natural science glasses". Students numbered 4-6 are instructed to watch the video from the perspective of humanistic science; students numbered 7-9 to watch the video from a social science perspective.

### Step 3 - Watch video

Students work with a case from their future practice - represented by a video. The video shows a clinical situation relevant for their education, in our case a normal birth; all students and teachers watch the video together in the classroom.

### Step 4 - Facilitate work in assigned groups

Book three classrooms: one for each scientific perspective. The natural science teacher and the students assigned the numbers 1-3 work together in one room, and the teachers of humanities and social sciences work with their groups, 4-6 and 7-9, respectively, in two other rooms. The groups work with the same epistemological questions ( Table 1) from the three different scientific perspectives. Thereby, the competence to transfer science into midwifery practice is facilitated in smaller groups together with the respective teachers. The length of the group work is 60 minutes.

The teacher’s role is to facilitate and guide students in the right direction and encourage students to answer the questions from their assigned scientific perspective. Students seem to benefit from smaller group and the teachers’ presence. It allows them to build courage to speak up even if they are unsure of the accuracy of their contribution and moreover, the teacher can give immediate feedback and encourage academic reflection.

Students create a shared document in each group to use as a background for their presentations. After the workshop, the documents are made available for the whole class.

### Step 5 - Make room for student preparation of presentation

Within all three scientific groups, students divide into three smaller groups, meaning that all students assigned number 1 are in one group, all assigned number 2 in another group and so on. They work for 20 minutes without teacher facilitation to prepare a short presentation based on their shared document. To prepare the presentation in this manner is helpful especially for weaker students who can get a chance to repeat and clarify. In the presentation, students reflect on the significance of their scientific perspective and assess and discuss their own understanding.

### Step 6 - Facilitate presentation of the three perspectives

Reorganize the groups so that there is one group of students from natural science, one from humanistic science and one from social science in each classroom (see Figure 1). In turns, the groups present their reply to the questions to the other students; thus, students are presented for the two perspectives with which they did not work themselves. All students participate actively as they verbalize their understanding of the perspective and engage in discussion of other perspectives. Students who tend to be passive during group work benefit especially from this part. The teacher’s role is to organize the presentations and participate in discussions to correct misunderstandings and encourage active peer interaction in the group. This step is scheduled for 45 minutes.

### Step 7 - Evaluate the workshop

Meet with all students to do a short evaluation of the process and the learning achieved. In our experience, students find the workshop very useful to enhance their understanding of the basic differences between natural sciences, humanities and social sciences. They begin to understand how these scientific perspectives broaden their perspective on midwifery practice. They often state that this is the first time that they have seen the relevance of both theory of science and clinical practice.

## Facilitation of transfer of epistemological knowledge to the clinical part of the education

According to the curriculum of the Program of Midwifery in Denmark, knowledge from theory of science must be related to learning situations in practice ( Ministry of Education, 2009). In the workshop, the video presents a clinical situation and therefore brings practice into theory. By referring to the epistemological goals for clinical practice, theory is brought into practice.

In clinical learning situations, transfer of knowledge happens during clinical teaching, meetings in the clinic, reflections in seminars and during supervisions. Questions exploring epistemological knowledge have been co-created with educators from clinical practice to facilitate knowledge transfer. For example: “How do you use knowledge from natural, social or humanistic sciences in your clinical assignment?” or “Where do you see elements of natural, social or humanistic sciences in your portfolios?”.

## Conclusion

This teaching approach/project demonstrates the relevance and equal worth of all epistemological perspectives in healthcare educations and the healthcare sector. The didactic design of the workshop promotes and facilitates students’ comprehension of how different scientific approaches are relevant to midwifery practice. The presence and cooperation between teachers create a common approach to epistemology and demonstrates acceptance of the equality and importance of all perspectives. In the group work, the teacher’s presence facilitates the student’s work with a specific scientific perspective, and co-learning among students is established. The students find that the teacher’s presence is important as it enables them to adjust their discussions immediately, which is specifically relevant when a level of philosophical thinking must be exemplified in a practical case.

As former mentioned, this workshop has been performed in different countries at different levels from bachelor students in Denmark to nurses in Norway and PhD students in Australia. The positive receipt of this workshop supports our confidence in this model for teaching epistemology and the importance of sharing this knowledge.

## Take Home Messages

• It is possible to improve the outcome of teaching epistemology.
• Working with a video from the students’ clinical practise connects theory and practise.
• When a learning situation is complex, teachers’ active participation facilitates learning.

## Notes On Contributors

**Ingrid Jepsen,** Ph.D. 2017 award. Master of Public Health Registered Midwife and Lecturer at Department of Midwifery at UCN. ORCID ID: https://orcid.org/0000-0002-7897-7036

**Lene Toxvig,** Master’s degree in Health and Humanities, Registered Midwife and Lecturer at the Department of Midwifery at UCN. ORCID ID: https://orcid.org/0000-0002-8579-3681

**Annegrethe Nielsen**, Ph.D. award 2005, Master of Arts. Former lecturer at the midwifery department at UCN. Employed at Nursing University College Copenhagen.
