# Supplementary material for: Teaching Epistemology – Workshop as a Method for making Epistemology Relevant to Students
Source: MedEdPublish (2016). 2021 May 22;10:140. [Version 1] doi: 10.15694/mep.2021.000140.1 (PMC10939543; doi:10.15694/mep.2021.000140.1)
Supplement: Peer review history

# Reviewer report: Reviewer response for version 1

**DOI:** 10.21956/mep.18938.r27091

**Published:** 2021-06-01

**Authors:** BALAJI ARUMUGAM

**Affiliations:** 1 TAGORE MEDICAL COLLEGE AND HOSPITAL

This review has been migrated. The reviewer awarded 4 stars out of 5 Thanks for the invite to review this article. Wonderfully executed new educational tool, workshop as a method to teach epistemology. Epistemology, the philosophical study of the nature, origin, and limits of human knowledge. The term is derived from the Greek epistēmē (“knowledge”) and logos (“reason”), and accordingly the field is sometimes referred to as the theory of knowledge.There are three main examples or conditions of epistemology: truth, belief and justification.The topic Epistemology is a tougher topic to teach and make the learner to understand and immerse themselves in the field. The method that the authors have adapted is a seven step well described workshop method for the variety of learners belonging to several specialties. Video demonstrations and then the followed discussions were well documented step by step. In the step 7, the evaluation of the workshop was expressed by the learners as this is the first time that they have seen the relevance of both theory of science and clinical practice.I congratulate the entire team who participated, conducted and reviewed this educational tool and thanks for sharing your experience. Suggestions: The author could have evaluated with the simple feedback questionnaire and could have presented the learners perspective more comprehensively in a tabular columns or as quotes. It would have become a research article.

Reviewer Expertise:

NA

No decision status is available

# Reviewer report: Reviewer response for version 1

**DOI:** 10.21956/mep.18938.r27090

**Published:** 2021-05-30

**Authors:** Helena Filipe

**Affiliations:** 1 Hospital of the Armed Forces/PL-EMGFA

This review has been migrated. The reviewer awarded 5 stars out of 5 Heartfelt acknowledgement for the opportunity to review this interesting and meaningful article. Kudos to the Authors for bringing to mind the relevant topic.Authors start by setting a well evidence-based floor to describe their study and define clearly its purpose. The educational format to approach research literacy, usually viewed detached from practice and hence a challenge to facilitate and get learners attention was ingenious. The jigsaw small group discussions is an engaging active learning strategy, close to the real world strategic approach in working organisations. Learners leave their home groups/organisations, seeking to improve focused expertise at an external organisation or expert group to bring back to their home group or organisation, specific intellectual capital to disseminate and reinforce. The home group/organisation will thus grow diversified learning expertise. Learners were predisposed to understand and embrace epistemology underpinning research considering a tríade of scientific perspectives. After allowing learners for some initial curated self-study time, faculty with expertise in humanistic, in social and sciences in natural sciences lectured in and shared a multimedia resource focusing each of the several research perspectives. Faculty facilitated and enabled their learners to work on a structured assignment in small groups. Finally, learners were offered an opportunity to reinforce their new learning by sharing their findings among each other in their home groups. Exceptionally conducted!A word of remark for the moment of debrief at the end of the workshop.The figure is visually appealing and facilitates understanding to the point of replication, the followed methodology. The table is well built and useful.The article reflects a consistent work with a wider scope in practice than research literacy, while incorporating a broader view into daily practice and encompassing self-regulation and reflection. This paper highlights, Authors´ (as Faculty) understanding and bridging the need of their learners, as healthcare professionals caring for human beings, of a holistic consideration of medical situations. It will impact practice, if professionals understand the nature of knowledge, how it is built, the scaffolding values, and the diverse perspectives as “There is more life in the spaces where different habitats meet” (1). Reference(1) Varpio L, MacLeod A. Philosophy of Science Series: Harnessing the Multidisciplinary Edge Effect by Exploring Paradigms, Ontologies, Epistemologies, Axiologies, and Methodologies. Acad Med. 2020 May;95(5):686-689. doi: 10.1097/ACM.0000000000003142. PMID: 31876567.

Reviewer Expertise:

NA

No decision status is available
